# The association of visceral adiposity index with the risk of kidney stone and kidney stone recurrence

**DOI:** 10.1186/s12882-023-03421-w

**Published:** 2023-12-12

**Authors:** Dan Liang, Chang Liu, Mei Yang

**Affiliations:** 1 Department of Endocrine, People’s Hospital of Chongqing Liang Jiang New Area, Chongqing, China; 2 The West China College of Medicine, Sichuan University, Chengdu, China; 3 School of Medicine, Nankai University, Tianjin, China

**Keywords:** VAI, Kidney stones, Kidney stones recurrence, NHANES

## Abstract

**Aim:**

Our aim in this study was primarily to assess the relationship between visceral adiposity index (VAI) and the risk of kidney stones and kidney stone recurrence in US adults.

**Methods:**

We used data from the National Health and Nutrition Examination Survey (NHANES) 2007–2014 for our analysis. VAI was calculated from waist circumference (WC), body mass index (BMI), triglycerides (TG), and high-density lipoprotein-cholesterol (HDL-C). Kidney stones and recurrence of kidney stones were obtained from questionnaire interview data. We used multivariate logistic regression analysis to explore the correlation between VAI and the risk of kidney stone and kidney stone recurrence. In addition, we performed subgroup analysis, interaction tests, and restricted cubic spline (RCS) analysis.

**Results:**

A total of 9886 participants were included in this study, with a prevalence of 9.24% for kidney stones and 2.97% for recurrence of kidney stones. The prevalence of kidney stones and kidney stone recurrence increased with higher quartiles of VAI. We observed a significantly positive correlation between VAI and the risk of kidney stone and kidney stone recurrence. Participants with the highest VAI quartiles had a 48% (OR: 1.48, 95%CI: 1.08–2.02) and 52% (OR: 1.52, 95%CI: 0.86–2.71) increased risk of kidney stones and kidney stone recurrence, respectively, compared to participants with the lowest VAI quartiles. Subgroup analysis and interaction tests demonstrated this positive association independent of different subgroup factors.

**Conclusion:**

Visceral fat accumulation may be associated with an increased risk of kidney stones and kidney stone recurrence.

**Supplementary Information:**

The online version contains supplementary material available at 10.1186/s12882-023-03421-w.

## Introduction

Kidney stones are a major public health problem worldwide and bring a heavy economic burden [1]. A UK cohort study found a rising trend in the cost of kidney stone treatment [2]. The prevalence of kidney stones has been reported to be approximately 10.1% among adults in the United States, and the prevalence of stones is significantly higher in men than in women [3]. In Europe, the prevalence of kidney stones is around 5%-14% [4, 5]. Besides, the recurrence rate of kidney stones within five years is about 50% [6]. Patients with kidney stones have a significantly increased risk of end-stage renal disease and chronic kidney disease compared to patients without kidney stones, even if the patient has only one episode of kidney stones [7]. Patients with recurrent symptomatic kidney stones have a higher risk of end-stage renal disease [8].

Obesity is also a global public health problem that threatens the health of all humans, with the prevalence of obesity increasing, with nearly 600 million adults reported to be obese worldwide in 2015 [9]. In addition, it is expected that by 2030, nearly 50% of U.S. adults will be obese [10]. Obese individuals are reported to be at greater risk of kidney stones, and the risk of kidney stones is further increased when obesity is combined with metabolic syndrome [11]. Another study that evaluated the association between kidney stones and obesity indicators found that body mass index (BMI), waist circumference (WC), waist-to-height ratio (WHtR), and waist-to-hip ratio (WHR), all of which are obesity-related indices, were significantly associated with an increased risk of kidney stones [12].

Traditional obesity-related indices such as BMI, WC, and WHtR are difficult to differentiate between subcutaneous and visceral fat accumulation. Visceral adiposity index (VAI), a sex-specific index based on WC, BMI, triglycerides, and HDL Cholesterol, can accurately identify and assess visceral adiposity function [13]. One study found that the positive predictive value of VAI for diabetes was significantly higher than that of BMI and WC [14]. VAI was also associated with a significantly increased risk of type 2 diabetes in older Chinese adults [15]. A 10-year follow-up study found that VAI was associated with a significant increase in cardiovascular disease prevalence [16]. Another study also confirmed that VAI was independently associated with an increased risk of angina, hypertension, and coronary atherosclerotic heart disease [17]. In hemodialysis patients, the investigators found that the predictive value of VAI for all-cause mortality was similar to that of WC and WHtR, while VAI was significantly better than WC in predicting the occurrence of cardiovascular events [18]. In addition, VAI can be used to predict renal function in patients with type 2 diabetes because it is significantly and positively correlated with the patient's urinary albumin level [19]. Another study also confirmed that VAI is closely associated with the manifestation of decreased renal function such as albuminuria. However, the association of VAI with the risk of kidney stones and kidney stone recurrence has been rarely reported before, so we used data from the National Health and Nutrition Examination Survey (NHANES) to assess the association between VAI and the risk of kidney stones and kidney stone recurrence.

## Materials and methods

### Study population

Data for our study were obtained from the National Health and Nutrition Examination Survey ( NHANES), a national cross-sectional survey study designed to assess the nutritional and health status of the U.S. population. The study recruited a highly representative sample due to the stratified, multi-stage, and probability sampling design approach of NHANES. All NHANES study protocols were approved by the National Center for Health Statistics (NCHS) Research Ethics Review Board and all participants signed written informed consent. A detailed description of the NHANES study and its data can be accessed online at https://www.cdc.gov/nchs/nhanes/.

**Fig. 1:**
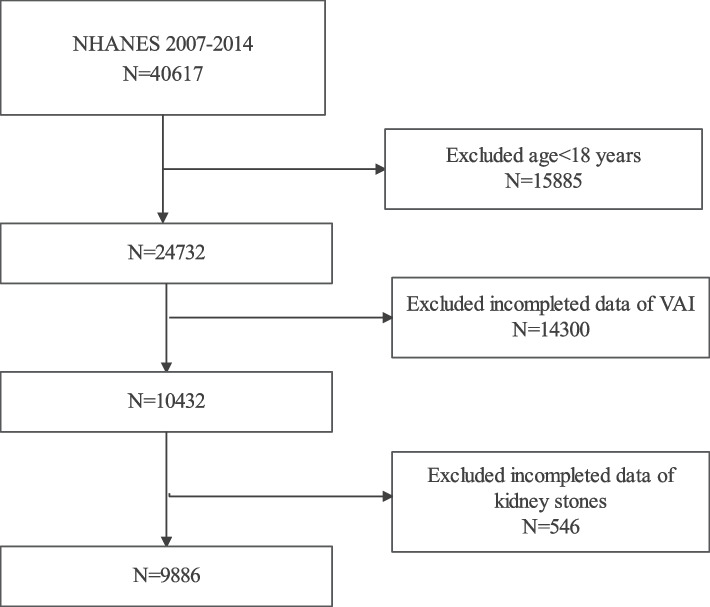
Flowchart of the sample selection from National Health and Nutrition Examination Survey (NHANES) 2007–2014

We obtained data from four consecutive cycles of NHANES 2007–2014, for which complete data on VAI, renal stones, and renal stone recurrence were available during the four consecutive survey cycles. A total of 40,617 subjects were initially included in our study, and after excluding participants < 18 years of age, and with missing data on VAI (*n* = 14,300) and kidney stones (*n* = 546), a total of 9886 participants were included in our final analysis (Fig. 1).

## Exposure and outcome definitions

VAI was designed as an exposure variable in our study. Visceral Adiposity Index (VAI) is a sex-specific index based on waist circumference (WC), body mass index (BMI), total triglycerides (TG), and high-density lipoproteins (HDL-C). The VAI was calculated as follows [13]: For males: VAI = WC/(39.68 + (1.88 × BMI)) × (TG/1.03) × (1.31/HDL-C); For females: VAI = WC/(36.58 + (1.89 × BMI)) × (TG/0.81) × (1.52/HDL-C). The units of TG and HDL are mmol/L, while the units of WC are cm. VAI can be used to assess visceral fat function. A higher VAI score represents a greater amount of visceral fat and the patient may be at higher risk for cardiovascular disease.

The incidence of kidney stones and the recurrence of kidney stones were designed as outcome variables. The status of kidney stones was assessed by the following two questions. These questions were "Have you ever had a kidney stone?" and "How many times have you passed a kidney stone?" Participants who answered yes were assumed to have kidney stones. Participants who had experienced two or more kidney stones were assumed to have a recurrence of kidney stones.

### Covariates

Our covariates included in these studies included age, sex, race, educational levels, BMI, physical activity, diabetes (DM) and hypertension. BMI was classified as < 25, 25–29.9, and ≥ 30 kg/m2, which corresponded to normal weight, overweight, and obese population for all participants. Hypertension was defined based on a self-reported diagnosis of hypertension, diastolic blood pressure ≥ 90 mmHg or systolic blood pressure ≥ 140 mmHg, or the use of anti-hypertensive medications [20]. DM was defined base on a self-reported diagnosis of diabete mellitus, 2-h plasma glucose ≥ 200 mg/dL in an oral glucose tolerance test, HbAlc ≥ 6.5%, use of oral hypoglycemic agents, or fasting glucose ≥ 126 mg/dL [21]. Physical activity (PA) status was collected through questionnaire interviews, which assessed PA in the past 30 days through questionnaires, mainly in the categories of walk, bicycle, work activity, and recreational activity. Staff calculated the MET minutes per week of each activity by multiplying the standard MET value of each activity by the total number of minutes per week of each activity. In accordance with previous studies [22], different groups were differentiated based on PA levels, where participants with PA < 150 MET-min/week were defined as Very low PA, participants with PA between 150–960 MET-min/week were defined as Low PA group, participants with PA between 960–1800 MET-min/week were defined as Medium PA, and those with PA > 1800 MET-min/week were defined as High PA group. All detailed measurement processes of these variables are publicly available at www.cdc.gov/nchs/nhanes/.

### Statistical analysis

All statistical analyses were performed according to Centers for Disease Control and Prevention (CDC) guidelines, with appropriate NHANES sampling weights applied and a complex multistage cluster survey design considered in the analysis. Continuous variables were presented as means with standard error, and categorical variables were presented as proportions. The difference between subjects grouped by VAI quartiles was evaluated by a weighted Student's t-test (for continuous variables) or weighted Chi-Square test (for categorical variables). Multivariable logistic regression was used in three different models to investigate the association between VAI and the risk of kidney stone and kidney stone recurrence. In Model 1, no covariates were adjusted. Model 2 was adjusted for age, gender, race, educational levels, and BMI (continuous variable). Model 3 was adjusted for age, sex, race, educational levels, BMI (continuous variable), PA, DM, and hypertension. We tested the correlation between VAI and the risk of kidney stone and kidney stone recurrence under different stratification factors by subgroup analysis. In addition, these stratification factors were also considered as potential modifiers, and an interaction term was added to test for heterogeneity between the subgroups. The analysis of potential nonlinear relationships employed restricted cubic spline (RCS) analysis, employing three piecewise points, to flexibly explore associations between VAI and the risk of kidney stone and kidney stone recurrence. All analyses were performed using R version 4.2.1 (The R Foundation). *P* < 0.05 was considered statistically significant.

## Results

### Participants' characteristics at baseline

A total of 9886 participants were included in this study with a mean age of 47.38 ± 0.31 years, of which 51.69% were females and 48.31% were males. The mean VAI index was 2.09 ± 0.04. The mean prevalence of kidney stones was 9.24% overall and increased with the increasing VAI quartiles (Quartile 1: 6.08%, Quartile 2: 8.57%, Quartile 3: 9.95%, Quartile: 12.40%). The recurrence rate of kidney stones in all participants was 2.97% and individuals with higher VAI quartile tended to have a higher risk of renal stone recurrence (Quartile 1: 1.78%, Quartile 2: 1.86%, Quartile 3: 3.78%, Quartile: 4.51%). Among the four VAI quartiles, statistically significant differences were found in age, total cholesterol, HDL-C, triglycerides, waist circumference, race, PIR, education, physical activity, BMI, alcohol consumption, smoking status, hypertension, and diabetes mellitus (*p* < 0.05). Compared to the lowest VAI quartile, participants in the increased VAI group had higher levels of total cholesterol, triglycerides, waist circumference, BMI, and lower levels of HDL-C, and were more likely to develop hypertension and diabetes. In addition, participants in the increased VAI group were more likely to be Mexican American and Non-Hispanic White, less physically active, and more likely to be current smokers and non-drinkers There was no statistical significance between quartiles in sex (*p* > 0.05) (Table 1).

**Table 1:** Baseline characteristics of the study population

	All participants	Q1	Q2	Q3	Q4	*P* value
Age (year)	47.38 (0.31)	44.31 (0.60)	46.75 (0.46)	48.82 (0.42)	49.67 (0.41)	** < 0.0001**
BMI (Kg/m^2)	28.72 (0.11)	25.55 (0.15)	27.75 (0.17)	29.91 (0.18)	31.72 (0.18)	** < 0.0001**
HDL-C (mmol/L)	1.39 (0.01)	1.76 (0.01)	1.46 (0.01)	1.29 (0.01)	1.06 (0.01)	** < 0.0001**
Triglyceride (mmol/L)	1.46 (0.02)	0.67 (0.01)	1.02 (0.01)	1.43 (0.01)	2.72 (0.05)	** < 0.0001**
Waist circumference (cm)	98.69 (0.27)	89.45 (0.39)	96.11 (0.43)	101.97 (0.35)	107.36 (0.46)	** < 0.0001**
VAI	2.09 (0.04)	0.63 (0.01)	1.17 (0.00)	1.89 (0.01)	4.70 (0.10)	** < 0.0001**
Gender (%)						0.23
Female	51.69 (0.02)	50.58 (1.20)	51.30 (1.16)	53.78 (1.06)	51.13 (1.24)	
Male	48.31 (0.02)	49.42 (1.20)	48.70 (1.16)	46.22 (1.06)	48.87 (1.24)	
Races (%)						** < 0.0001**
Mexican American	8.72 (0.01)	6.08 (0.63)	8.19 (1.06)	10.24 (1.20)	10.41 (1.10)	
Non-Hispanic Black	10.25 (0.01)	16.46 (1.34)	10.80 (0.88)	8.67 (0.83)	5.00 (0.54)	
Non-Hispanic White	68.29 (0.04)	64.91 (1.96)	68.36 (1.95)	68.15 (1.87)	71.78 (2.01)	
Others	12.74 (0.01)	12.55 (1.24)	12.66 (1.02)	12.94 (0.89)	12.81 (1.27)	
Educational levels (%)						** < 0.0001**
Less than 9th grade	6.05 (0.00)	4.07 (0.49)	5.43 (0.50)	6.78 (0.69)	7.95 (0.65)	
9-11th grade	12.13 (0.01)	9.27 (0.67)	11.21 (0.99)	12.89 (0.91)	15.20 (0.93)	
High school graduate	29.73 (0.01)	18.83 (1.10)	22.10 (1.26)	21.97 (1.22)	24.16 (1.44)	
Some college or AA degree	21.76 (0.01)	29.28 (1.23)	28.60 (1.14)	31.99 (1.49)	31.48 (1.40)	
College graduate or above	30.33 (0.01)	38.56 (1.67)	32.66 (1.66)	26.37 (1.57)	21.21 (1.36)	
Physical activity (%)						** < 0.0001**
Very low PA	24.16 (0.01)	17.49 (1.03)	21.77 (1.04)	26.69 (1.22)	30.79 (1.40)	
Low PA	21.24 (0.01)	20.09 (0.91)	21.13 (1.03)	22.03 (1.24)	21.72 (1.23)	
Medium PA	12.50 (0.00)	13.10 (0.87)	13.92 (0.72)	11.76 (0.67)	11.20 (0.87)	
High PA	42.10 (0.02)	49.32 (1.34)	43.18 (1.01)	39.52 (1.29)	36.29 (1.41)	
BMI (%)						** < 0.0001**
Normal weight	30.96 (0.01)	53.97 (1.24)	35.86 (1.53)	22.22 (1.21)	11.74 (0.87)	
Overweight	33.61 (0.01)	29.43 (1.06)	36.38 (1.37)	34.99 (1.31)	33.99 (1.13)	
Obesity	35.18 (0.01)	16.60 (0.84)	27.76 (1.26)	42.79 (1.24)	54.27 (1.29)	
DM (%)	15.69 (0.01)	7.34 (0.61)	10.63 (0.81)	18.07 (1.06)	26.88 (1.15)	** < 0.0001**
Hypertension (%)	37.22 (0.02)	24.57 (1.20)	34.10 (1.34)	39.67 (1.32)	50.69 (1.20)	** < 0.0001**
Kidney stone (%)	9.24 (0.01)	6.08 (0.55)	8.57 (0.90)	9.95 (0.64)	12.40 (0.93)	** < 0.0001**
Kidney stone recurrence (%)	2.97 (0.00)	1.78 (0.36)	1.86 (0.32)	3.78 (0.55)	4.51 (0.69)	** < 0.0001**

*BMI* Body mass index, *VAI* Visceral adiposity index, *PA* Physical activity

Continuous variables were expressed as means and standard errors, and categorical variables were expressed as percentages

### The association of visceral adiposity index and the risk of kidney stone

The association between VAI and the risk of kidney stones was indicated in Table 2. The authors observed that higher VAI was associated with an increased risk of kidney stones. In the crude model (Model 1), we observed a positive association between increased VAI and kidney stone risk. This positive correlation remained significant in Model 2 adjusted for age, sex, and race. In the fully adjusted model (Model 3), the positive correlation between VAI and kidney stone risk remained robust. The authors identified a 2% increase in the risk of kidney stones for each unit increase in VAI (OR = 1.02, 95%CI: 1.00–1.04). This positive association remained statistically significant when we considered VAI as quartiles. Participants in the highest quartile of VAI had a significant 41% increased risk of kidney stones compared to those in the lowest quartile of VAI (OR: 1.41, 95%CI: 1.06–1.87) (Table 2).

**Table 2:** The association between VAI and the risk of kidney stones

Kidney stone	OR (95%CI)		
	Model 1	Model 2	Model 3
VAI index	1.03 (1.01, 1.05), *p* = **0.01**	1.01 (1.00, 1.04), ***p* = 0.01**	1.02 (1.00, 1.04), ***p* = 0.04**
VAI index			
Quartile 1	Reference	Reference	Reference
Quartile 2	1.45 (1.10, 1.92), ***p* = 0.01**	1.24 (1.02, 1.66), ***p* = 0.04**	1.23 (1.02, 1.63), ***p* = 0.02**
Quartile 3	1.71 (1.32, 2.21), ***p* < 0.0001**	1.29 (1.17, 1.72), ***p* = 0.01**	1.26 (1.04, 1.68), ***p* = 0.02**
Quartile 4	2.19 (1.73, 2.77), ***p* < 0.0001**	1.49 (1.13, 1.98), ***p* = 0.01**	1.41 (1.06, 1.87), ***p* = 0.02**

*BMI* Body mass index, *PA* Physical activity, *DM* Diabetes

Model 1: No covariates were adjusted

Model 2: Age, gender and race, educational levels and BMI (continous variable) were adjusted

Model 3: Age, sex, race, education, educational levels, BMI (continous variable), PA, DM and hypertension were adjusted

**Fig. 2:**
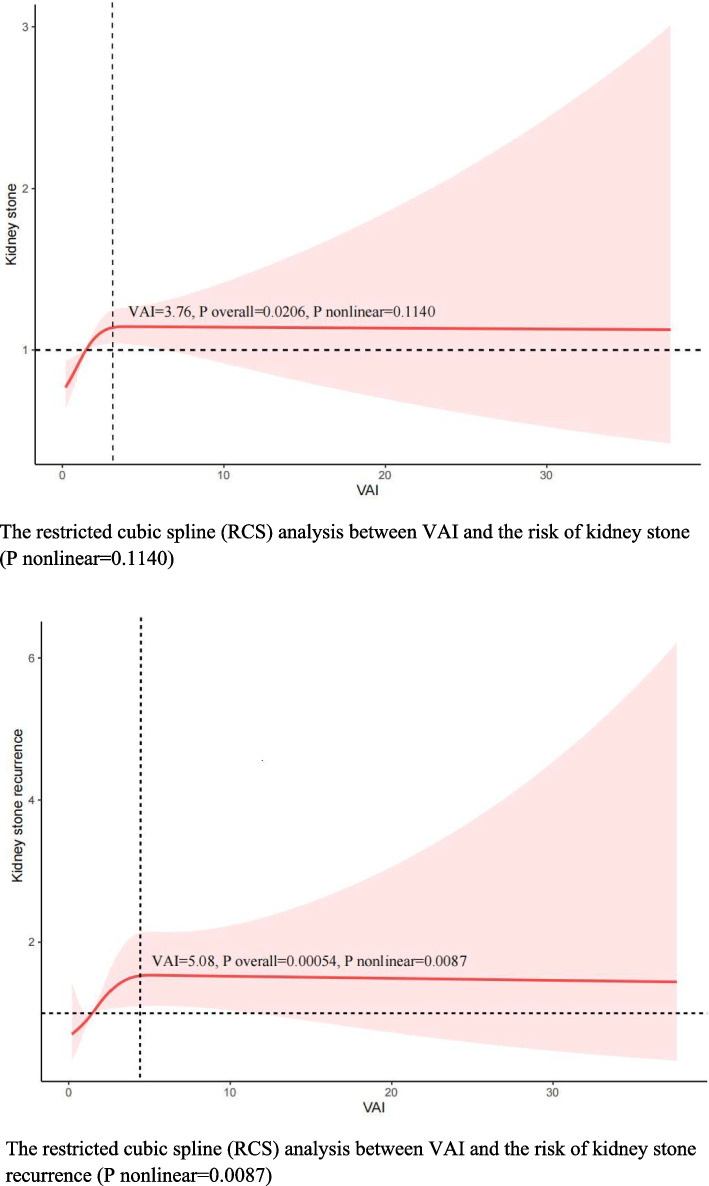
The restricted cubic spline (RCS) analysis between VAI and the risk of kidney stone and kidney stone recurrence

To further investigate whether there was a nonlinear relationship between VAI and kidney stones, we performed an RCS analysis. Our results revealed no nonlinear relationship between VAI and kidney stones (P nonlinear = 0.1140) (Fig. 2).

### The association of visceral adiposity index and the risk of kidney stone recurrence

For the risk of kidney stone recurrence, We also observed a positive association between VAI and the likelihood of kidney stone recurrence. A significant positive association between VAI and the risk of kidney stone recurrence was observed in Model 1 and Model 2 (Model 1: OR: 1.03, 95%CI: 1.01–1.06; Model 2: OR: 1.02, 95%CI: 1.00–1.04). After full adjustment, we observed that each 1-unit increase in VAI increased the risk of kidney stone recurrence by 2% (Model 3: OR: 1.02, 95%CI: 1.01–1.04).

When we treated VAI as quartiles, we could still observe a positive association between VAI and the risk of kidney stone recurrence. Subjects in the highest quartile had a significantly increased risk of kidney stone recurrence by 63% compared to subjects in the lowest VAI quartile (OR: 1.63, 95%CI: 1.43–2.76) (Table 3).

**Table 3:** The association between VAI and the risk of kidney stone recurrence

Kidney stone recurrence	OR (95%CI)		
	Model 1	Model 2	Model 3
VAI index	1.03 (1.01, 1.06), ***p* = 0.01**	1.02 (1.00, 1.04), ***p* = 0.02**	1.02 (1.01, 1.04), ***p* = 0.04**
VAI index			
Quartile 1	Reference	Reference	Reference
Quartile 2	1.05 (1.02, 1.76), ***p* = 0.01**	1.36 (1.15, 1.65), ***p* = 0.04**	1.18 (1.09, 1.55), ***p* = 0.04**
Quartile 3	2.17 (1.26, 3.75), ***p* = 0.01**	1.94 (1.08, 3.49), ***p* = 0.03**	1.56 (1.29, 3.03), ***p* = 0.01**
Quartile 4	2.61 (1.72, 3.96), ***p* < 0.0001**	2.22 (1.40, 3.51), ***p* < 0.001**	1.63 (1.43, 2.76), ***p* < 0.001**

*BMI* Body mass index, *PA* Physical activity, *DM* Diabetes

Model 1: No covariates were adjusted

Model 2: Age, gender and race, educational levels and BMI (continous variable) were adjusted

Model 3: Age, sex, race, education, educational levels, BMI (continous variable), PA, DM and hypertension were adjusted

In addition, we also explored the non-linear relationship between VAI and the risk of kidney stone recurrence by RCS analysis. Our results showed that the relationship between VAI and kidney stone recurrence was nonlinear (P nonlinear = 0.0087) (Fig. 2).

### Subgroup analysis

**Fig. 3:**
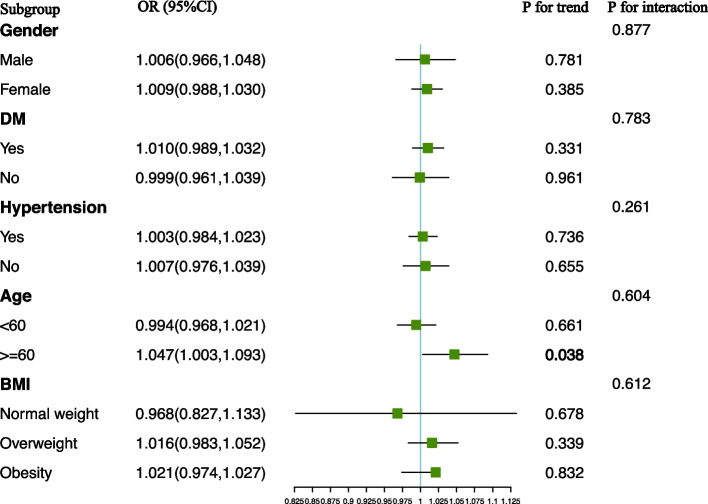
Subgroup analysis for the association between VAI and kidney stones

For the correlation between VAI and kidney stones, we observed a positive association among participants stratified by age. Each unit increase in VAI was significantly associated with a 4.7% increase in the risk of kidney stones in participants aged 60 years or older (OR: 1.047, 95% CI: 1.003–1.093). However, in the interaction test, we did not find an effect of age on the correlation between VAI and kidney stones (P for interaction = 0.604) (Fig. 3).

**Fig. 4:**
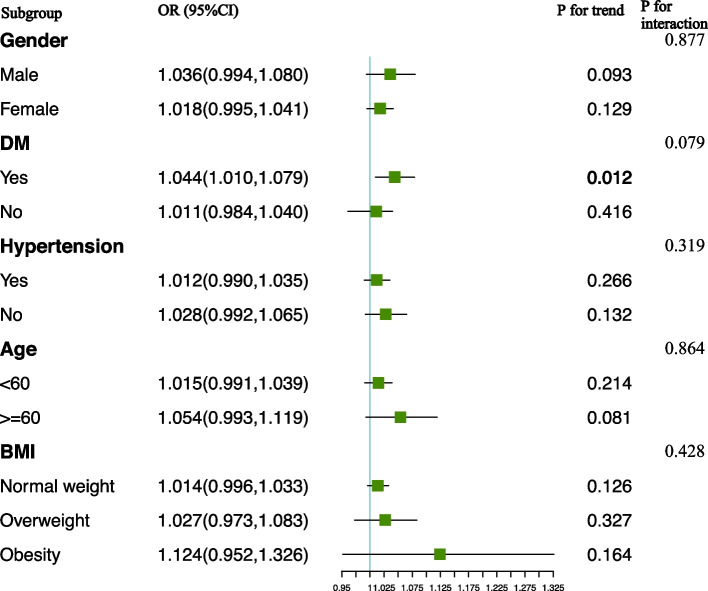
Subgroup analysis for the association between VAI and kidney stone Recurrence

In addition, we evaluated the association between VAI and the risk of renal stone recurrence by subgroup analysis. A significant association was observed in participants who had diabetes (OR: 1.044, 95%CI: 1.010–1.079). Interaction terms were also used to test the heterogeneities among each subgroup, and our results showed that no significant difference was revealed among diabetes status (P for interaction = 0.079), indicating that this association was not dependent on diabetes status (Fig. 4). Furthermore, no significant differences were indicated by the interaction test across other stratifications, suggesting that this positive association between VAI and kidney stone recurrence was not significantly influenced by gender, age, hypertension, and BMI, and could be applied in a variety of population settings.

### Multivariate logistic regression models of kidney stone and kidney stone recurrence

Age, gender, race, educational levels, BMI, physical activity, and hypertension remained significantly associated with the risk of kidney stones in the fully adjusted model (Supplemental Table 1). We observed that each 1 unit increase in age was associated with a 2% increase in the risk of kidney stones (OR: 1.02, 95%CI: 1.01–1.05). Men had a 29% increased odds of kidney stones compared to women (OR: 1.29, 95%CI: 1.08–1.53). Compared to Mexican American, participants who were non-Hispanic Black and non-His panic White had a 45% lower risk (OR: 0.55, 95%CI: 0.43–0.72) and a 43% elevated risk (OR: 1.43, 95%CI: 1.13–1.82) of kidney stones, respectively. Compared with those who had a high PA, the odds of kidney stones were elevated by 6% and 13% in participants who had a low PA (OR = 1.06, 95%CI: 1.02–1.36) and very low PA (OR = 1.13, 95%CI: 1.01–1.40), respectively. Per unit increase in BMI, the odds of kidney stones were increased by 3%. Besides, compared with participants without hypertension, those with hypertension had a 26% increased risk of kidney stones (OR = 1.26, 95%CI: 1.01–1.57).

We also analyzed the possible factors associated with the risk of kidney stone recurrence by multifactorial analysis in Supplemental Table 2. Compared with female participants, male participants had a 59% increased risk of kidney stone recurrence(OR:1.59, 95%CI: 1.21–2.09). Compared with Mexican American, the risk of kidney stone recurrence in non-Hispanic White and non-Hispanic Black were lowered by 65% (OR = 0.35, 95%CI: 0.17–0.75) and elevated by 78% (OR = 1.78, 95%CI: 1.09–2.90), respectively. Per unit increase in BMI, the odds of kidney stone recurrence were increased by 6% (OR = 1.06, 95%CI: 1.03–1.09). The odds of kidney stone recurrence were increased by 6% and 45% in hypertension (OR = 1.06, 95%CI: 1.02–1.18) and diabetes (OR = 1.45, 95%CI: 1.05–2.20) populations compared with their counterparts.

## Discussion

This study, which recruited 9886 participants, showed that participants with higher VAI were more likely to develop kidney stones and recurrence of kidney stones. The results of our subgroup analysis and interaction tests also illustrated that this positive association was independent of sex, age, BMI, hypertension, diabetes, and smoking status. Management targeting visceral fat distribution may reduce the incidence of kidney stones, and clinicians should pay more attention to this indicator.

Although fewer studies have reported the association between VAI and the risk of kidney stones and recurrence of kidney stones, previous studies have described the relationship between VAI and kidney function. One study found that in older adults, VAI was associated with an increased risk of chronic kidney disease (CKD)-related events such as decreased glomerular filtration rate and rapid kidney function decline [23]. VAI can be a good predictor of CKD, with a prediction rate of 77% [24]. Excessive visceral fat deposition in patients with CKD increases the risk of all-cause mortality [25]. VAI was also independently associated with a significantly higher urinary albumin creatinine ratio in patients with prediabetes [26]. VAI also plays an important value in predicting the development of end-stage renal disease in patients with type 2 diabetes [27, 28]. Kim et al. found that visceral fat accumulation could be a better predictor of CKD severity than BMI, regardless of the patient's gender [29]. Another study in Korean adults showed a positive association of VAI with CKD incidence in men only, and this association was no longer significant in women [30]. Otunctemur et al. suggested that VAI levels are associated with the progression of renal cell carcinoma [31]. Fukuhara et al. also revealed VAI levels in patients after renal transplantation, with increasing visceral fat accumulation as the time after transplantation increased [32].

One study found that a BMI > 30 kg/m2 was independently associated with an increased risk of kidney stone disease [33]. An almost twofold increased risk of kidney stones was also observed in pediatric kidney stone patients with a BMI > 30 kg/m2 compared to those with a BMI in the normal range [34]. A US study found a 3% prevalence of kidney stones in participants without comorbid metabolic syndrome and a significant increase in the prevalence of kidney stones in participants with comorbid metabolic syndrome (hyperlipidemia, hypertriglyceridemia, hyperglycemia, hypertension, and abdominal obesity), with a 9.8% prevalence of kidney stones in those with five comorbid metabolic syndrome features [35]. Significant dyslipidemia has been identified in patients with kidney stones [36]. Another study found that dyslipidemia was associated with altered chemical composition in the urine and this chemical composition played a key role in stone formation, such as patients with high triglycerides exhibited increased uric acid excretion and significantly lower urinary pH, those with high total cholesterol had significantly higher urinary calcium levels, and patients with reduced high-density lipoprotein (HDL) showed significantly higher levels of oxalic acid and uric acid in their urine [37]. Mosli et al. also analyzed the composition of kidney stones in overweight and obese patients and found that their stones were predominantly composed of calcium oxalate and uric acid [38]. Hypercalciuria and hyperoxaluria were identified as high risk factors for calcium stone formation, while excessive uric acid excretion and decreased urinary pH were the main abnormal factors contributing to uric acid stone formation [39]. Furthermore, in obese patients, the accumulation of fat in proximal tubular cells reduced their ability to secrete ammonia, resulting in urine acidification, which could contribute to stone progression [40] Significantly increased levels of osteopontin and biomarkers associated with inflammatory responses and accumulation of pro-inflammatory macrophages were also observed in animal experiments in high-fat-fed mice, ultimately leading to crystal and mineral deposition in the tubular lumen of the kidneys and resulting in the progression of kidney stones [41]. These mice also exhibited a notable increase in oxalic acid levels in their urine [41]. In contrast, the administration of statin treatment in high-fat diet-fed mouse models led to increased renal osteopontin expression and reduced deposition of calcium oxalate stones [42]. The strong association between dyslipidemia, one of the important indicators for calculating the visceral fat accumulation index, and the progression of kidney stones explains the increased risk of kidney stones due to high VAI levels. However, Besiroglu et al. discovered a significantly elevated risk of urolithiasis in patients with high triglyceride (TG) and low high-density lipoprotein (HDL) levels [43]. Furthermore, the authors observed a robust correlation between any lipid profile disorders and the risk of urolithiasis. However, the association between TG and urolithiasis was found to be more consistent when compared to the association between HDL and urolithiasis. Cai et al. observed that non-stone-forming individuals in the TG group had increased urinary oxalate excretion, while those in the high TC group exhibited increased urinary calcium excretion. These constituents are known to be associated with urinary lithogenesis [44]. In contrast, individuals in the high low-density lipoprotein (LDL) group had decreased urinary calcium and oxalate excretion, whereas those in the low high-density lipoprotein (HDL) group displayed increased urinary citrate and magnesium excretion, which are protective factors against stone formation [44]. The physiological mechanisms underlying why specific components of dyslipidemia have paradoxical effects on urinary changes related to stone formation remain unclear at present. Future studies should also prioritize investigating potential connections between lipid metabolic profiles and different types of stone formations. Additionally, well-designed prospective randomized controlled trials or cohort studies are required to provide a more comprehensive understanding of the causal relationship between dyslipidemia and urolithiasis.

This study had several strengths. First, this study was based on data from NHANES, and because NHANES was a national population-based sample survey, the sample size obtained was fully representative. Second, we adjusted for confounding covariates to reduce confounding bias and to make our results more reliable.

Our study also had some limitations. First, the type of our study was a cross-sectional study, and the design of this type of study prevented us from obtaining a clear causal relationship between VAI and kidney stones. Second, although we have adjusted for several potential covariates, we cannot ensure that we have excluded all covariates that could lead to confounding, such as the use of some drugs. Finally, because the NHANES sample was specific to the US population only, our results may not be generalizable to the world.

## Conclusion

Elevated VAI showed a positive association with the risk of kidney stones and kidney stone recurrence. Our present study highlighted the importance of visceral fat accumulation management in patients at risk for kidney stones. However, further large prospective studies are needed to confirm the causal relationship between VAI and kidney stones.

### Supplementary Information

**Additional file 1: Supplemental Table 1. **Multivariate logistic regression models of kidney stones.

**Additional file 2: Supplemental Table 2. **Multivariate logistic regression models of kidney stone recurrence.

## Data Availability

Data described in the manuscript, codebook, and analytic code will be made publicly and freely available without restriction at www.cdc.gov/nchs/nhanes/.
