# Supplementary material for: The association of visceral adiposity index with the risk of kidney stone and kidney stone recurrence
Source: BMC Nephrol. 2023 Dec 12;24:368. doi: 10.1186/s12882-023-03421-w (PMC10717979; doi:10.1186/s12882-023-03421-w)
Supplement: Supplementary file 1 — Additional file 1: Supplemental Table 1. Multivariate logistic regression models of kidney stones. [file 12882_2023_3421_MOESM1_ESM.doc]

**Supplemental Table 1 Multivariate logistic regression models of kidney stones.**

| Variables | Kidney stones  OR (95% CI) | P value |
| --- | --- | --- |
| VAI index | 1.02 (1.00, 1.04) | **0.04** |
| Age (year) | 1.02 (1.01, 1.05) | **<0.0001** |
| Male (verus female) | 1.29 (1.08, 1.53) | **0.005** |
| Races (verus Mexican American) |  |  |
| Non-Hispanic Black | 0.55 (0.43, 0.72) | **<0.0001** |
| Non-Hispanic White | 1.43 (1.13, 1.82) | **0.004** |
| Other races | 1.15 (0.85, 1.54) | 0.36 |
| Educational levels (verus <9th grade) |  |  |
| 9-11th grade | 1.11 (0.78, 1.58) | 0.54 |
| High school graduate | 1.08 (0.75, 1.54) | 0.69 |
| Some college or AA degree | 1.07 (1.75, 1.53) | 0.69 |
| College graduate or above | 0.96 (0.66, 0.98) | **0.03** |
| BMI | 1.03 (1.02, 1.05) | **<0.0001** |
| Physical activity (verus High PA) |  |  |
| Medium PA | 1.01 (0.75, 1.34) | 0.98 |
| Low PA | 1.06 (1.02, 1.36) | **0.03** |
| Very Low PA | 1.13 (1.01, 1.40) | **0.02** |
| DM (verus No) | 1.21 (0.97, 1.53) | 0.09 |
| Hypertension (verus No) | 1.26 (1.01, 1.57) | **0.04** |
