# Supplementary material for: The association of visceral adiposity index with the risk of kidney stone and kidney stone recurrence
Source: BMC Nephrol. 2023 Dec 12;24:368. doi: 10.1186/s12882-023-03421-w (PMC10717979; doi:10.1186/s12882-023-03421-w)
Supplement: Supplementary file 2 — Additional file 2: Supplemental Table 2. Multivariate logistic regression models of kidney stone recurrence. [file 12882_2023_3421_MOESM2_ESM.doc]

**Supplemental Table 2 Multivariate logistic regression models of kidney stone recurrence.**

|  | Kidney stones  OR (95% CI) | P value |
| --- | --- | --- |
| VAI index | 1.02 (1.01, 1.04) | **0.04** |
| Age (year) | 1.02 (1.01, 1.03) | **<0.001** |
| Male (verus female) | 1.59 (1.21, 2.09) | **0.001** |
| Races (verus Mexican American) |  |  |
| Non-Hispanic Black | 0.35 (0.17, 0.75) | **0.01** |
| Non-Hispanic White | 1.78 (1.09, 2.90) | **0.02** |
| Other races | 1.55 (0.85, 2.83) | 0.15 |
| Educational levels (verus <9th grade) |  |  |
| 9-11th grade | 1.96 (1.15, 3.32) | **0.01** |
| High school graduate | 2.23 (1.41, 3.53) | **0.001** |
| Some college or AA degree | 0.70 (0.52, 0.84) | **0.04** |
| College graduate or above | 0.89 (0.71, 0.98) | 0.10 |
| BMI | 1.06 (1.03, 1.09) | **<0.001** |
| Physical activity (verus High PA) |  |  |
| Medium PA | 1.03 (0.64, 1.66) | 0.91 |
| Low PA | 1.26 (0.59, 1.41) | 0.67 |
| Very Low PA | 1.24 (1.02, 1.50) | **0.04** |
| DM (verus No) | 1.45 (1.05, 2.20) | **0.03** |
| Hypertension (verus No) | 1.06 (1.02, 1.18) | **0.04** |
